# Clinical utility of cryptococcal antigen detection in transthoracic needle aspirate by lateral flow assay for diagnosing non-HIV pulmonary cryptococcosis: A multicenter retrospective study

**DOI:** 10.1097/MD.0000000000030572

**Published:** 2022-09-16

**Authors:** Qun Hu, Xiaohua Li, Xiao Zhou, Chunlei Zhao, Caixia Zheng, Liyu Xu, Zizi Zhou

**Affiliations:** a Department of Pulmonary and Critical Care Medicine, South China Hospital of Shenzhen University, Shenzhen, China; b Department of Pulmonary and Critical Care Medicine, Fuzhou first hospital, Fujian Medical University, Fuzhou, China; c Department of Pulmonary and Critical Care Medicine, Fuzhou General Clinical Medical College, Fujian Medical University, Fuzhou, China; d Medical Imaging Center, Fuzhou General Clinical Medical College, Fujian Medical University, Fuzhou, China; e Department of imaging Medicine, Fuzhou First Hospital, Fujian Medical University, Fuzhou, China; f Department of Cardiothoracic Surgery, Shenzhen University General Hospital, Shenzhen, China.

**Keywords:** lateral flow immunoassay, non-HIV, pulmonary cryptococcosis, transthoracic needle aspiration

## Abstract

Lateral flow immunoassay (LFA) detection of cryptococcal capsular polysaccharide antigen (CrAg) is reported to be the most rapid and convenient laboratory method for diagnosing cryptococcosis. Its clinical diagnostic use, however, is not well studied. We retrospectively analyzed the data from 97 patients with suspected pulmonary cryptococcosis (PC) at 2 tertiary care centers. CrAg in both serum and lung aspirate specimens were examined by LFA. We divided the patients who were diagnosed with PC into group I, patients positive for CrAg in both the serum and lung aspirate, and group II, patients positive for CrAg in the lung aspirate but not in the serum. We analyzed the differences in imaging distribution, morphological characteristics, and concomitant signs between the 2 groups. Of all 97 patients, 47 were diagnosed with PC. Lung aspirates were positive for CrAg in 46/47 patients with PC (sensitivity 97.9%, specificity 100%, positive predictive value = 100%, negative predictive value = 98%). There were no false positive results in the noncryptococcosis patients, revealing a diagnostic accuracy of 99%. Serum CrAg tests were positive in 36/47 patients with PC (sensitivity 76.6%, specificity 100%, accuracy 88.7%, positive predictive value = 100%, negative predictive value = 82%). Chest imaging data showed a statistically significant greater number of single lesions in group II than in group I (*P* < .05). More lesions accompanied by halo signs were showed in group I (*P* < .01), whereas more accompanied by pleural stretch signs were found in group II (*P* < .01). The LFA-positive rate of CrAg in lung aspirate samples was higher than that of the serum samples, especially in patients with single pulmonary lesion or in those accompanied by pleural stretch. The direct measurement of CrAg in lung aspirate is a rapid, useful alternative diagnostic method for PC confirmation.

## 1. Introduction

Cryptococcal disease (cryptococcosis) is caused by *Cryptococcus* spp., which belong to basidiomycetes. The most common clinical manifestation of cryptococcosis is pulmonary cryptococcosis (PC).^[1]^ Worldwide incidence of PC infection has been increasing over the past decades in both patients with and without HIV.^[2–5]^ The prevalence of cryptococcosis in patients without HIV is being given increasing attention.^[6–8]^

PC is often misdiagnosed as tuberculosis or lung cancer because of similar clinical features and radiological characteristics. Therefore, diagnostic tools specific for cryptococcosis are necessary. It has been reported that serum cryptococcal capsular polysaccharide antigen (CrAg) test is both rapid and sensitive in diagnosing PC, as histology and culture are both important for confirming PC.^[9]^ Multiple assays have been developed; among these, the FDA has approved lateral flow assay (LFA) (IMMY, Norman, OK) for detection of CrAg. Some studies have been conducted to evaluate the performance of LFA methods. Serum and cerebrospinal fluid LFA has already been shown highly accurate for the diagnosis of cryptococcosis in patients at risk,^[10,11]^ while LFA in urine can be a promising sample screening tool for early cryptococcosis diagnosis.^[11]^

A previous study reported that, in non-HIV hosts, the rate of cryptococcal glucuronoxylomannan positive bronchoalveolar lavage fluid (BALF) samples was higher than the rate for serum samples, indicating that testing for the presence of the cryptococcal glucuronoxylomannan antigen in BALF specimens might contribute to the early PC diagnosis.^[12]^ It was reported that the direct measurement of cryptococcal antigen in the lung aspirate latex agglutination test (LA) can be a rapid and useful test for the diagnosis of PC.^[13]^ The CrAg LFA offers a number of advantages over LA, including enhanced sensitivity for detection of CrAg and a relatively rapid 15-min turnaround time; in addition, the assay does not require pronase pretreatment of serum samples.^[14–16]^ There are no reports to evaluate the diagnostic value of LFA in lung biopsy samples for PC yet, especially in patients without HIV. Thus, a multicenter, retrospective study was performed to investigate the diagnostic accuracy of the direct detection of CrAg by LFA in lung aspirates obtained by computed tomography (CT)-guided transthoracic aspiration.

## 2. Methods

### 2.1. Patients

This multicenter retrospective study was performed in an HIV-negative Chinese population by reviewing the medical records of 97 patients between April 2014 and March 2017 at the Affiliate Fuzhou City First Hospital of Fujian Medical University (Fuzhou, Fujian, China) and Fuzhou General Clinical Medical College of Fujian Medical University (Fuzhou, Fujian, China).

### 2.2. Radiological assessment

All the patients enrolled in this study underwent CT examinations. A radiologist and a respiratory physician independently assessed each patient’s CT scans and recorded the distribution, morphological features, and concomitant signs of the lesions. The distribution types included unilateral single, unilateral multiple, and bilateral multiple lesions. The morphological features included ground-glass opacity, nodules or masses, consolidation infiltrates, and mixed types. The concomitant signs include halos, cavitation or vacuoles, pleural traction, burrs, or other signs.

### 2.3. Detection of CrAg by LFA

The lesions were sampled using CT-guided percutaneous transthoracic aspiration biopsy after the assessment of pulmonary infiltration. The aspirate was obtained from CT window-available nodules or infiltrates and was immediately submitted for histopathological examination, cryptococcal antigen testing, and microbiological examination, including cultures for aerobes and anaerobes, mycobacteria, fungi, and cytological examination, which included Gram stains and acid-fast stains. If the aspirate volume was <0.1 mL, it was diluted with 1 mL of normal saline (dilution factor, ≥10-fold)^[12]^ and the examinations were then conducted as described above. All these patients were tested for the presence of CrAg in both their serum and lung aspirate specimens by LFA using the IMMY Cryptococcal Lateral Flow Assay (Immuno- Mycologics, Inc., Norman, OK) according to manufacturer instructions.

### 2.4. Definition of a pulmonary cryptococcosis diagnosis

The diagnosis of PC was confirmed by either a positive BALF culture or the presence of *C. neoformans* in the histopathological or cytological specimens, including transbronchial lung biopsies and CT-guided percutaneous lung biopsies.^[17]^ Patients with serum positive for CrAg but with negative culture, histopathologic, or cytologic examinations were defined as clinically diagnosed. Those with positive lung aspirate antigen results but negative cultures, histopathologic, cytologic, and serum CrAg results were not given a definitive diagnosis.

### 2.5. Predisposing conditions

The predisposing conditions of these patients with non-HIV were corticosteroid or immunosuppressive therapy, malignancy, and rheumatologic disease.^[18]^ Patients with no identifiable predisposing conditions who had negative testing for CD4 ^+^ lymphocytopenia and immunoglobulin deficiency were classified as having no underlying disease.

### 2.6. Statistics

The IBM SPSS ver. 22.0 (IBM Corp., Armonk, NY) was used to analyze the data. The Chi-squared test was used to compare categorical variables. Statistical significance was established with 2-sided *P* values <.05.

## 3. Results

### 3.1. Patient characteristics

**Figure 1.**
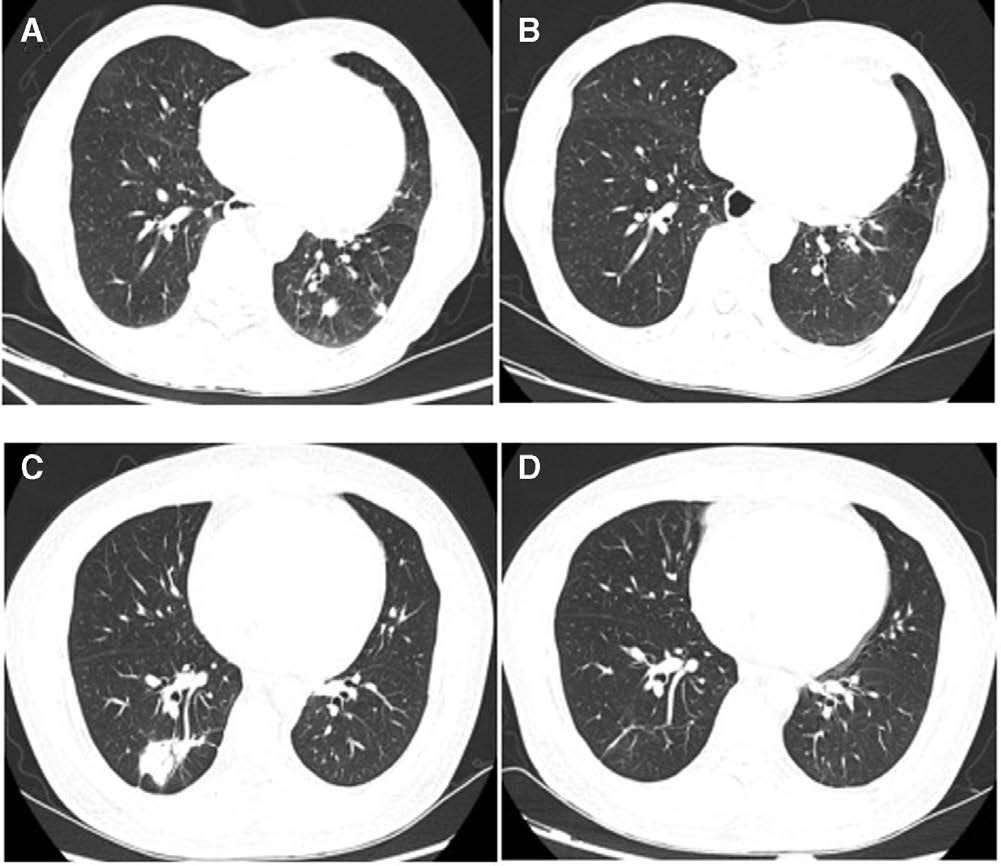
Chest CT scan of possible patients with pulmonary cryptococcosis. (A) Upon physical examination, a 78-year-old smoking male was found with multiple nodules in the left lower lung by a chest CT. The left lower lung lesion underwent CT-guided percutaneous transthoracic aspiration biopsy and the histopathological results indicated inflammatory granuloma, negative acid-resistant staining, PAS staining, and PAM staining. The results of serum CrAg LFA tests were shown negative, but lung aspirate CrAg LFA tests were positive. (B) Fluconazole was administered for 8 months, and the lesions were almost absorbed in this 78-year-old possible PC case. (C) A 38-year-old nonsmoking male had a cough for 3 weeks and was treated with levofloxacin hydrochloride tablets (500 mg/d, taken orally) for > 1 week, but his clinical condition did not improve. A CT-guided lung biopsy was performed on a right lower lung lesion. The histopathological results indicated inflammatory granuloma, negative acid-resistant staining, PAS staining, and PAM staining. Serum was negative for CrAg, but lung aspirates were positive. (D) Fluconazole was administered for 6 months and the lesions were absorbed in this 38-year-old possible PC case. CrAg = cryptococcal capsular polysaccharide antigen, CT = computed tomography, LFA = lateral flow immunoassay, PAM = periodic acid-silver methenamine, PAS = periodic acid-Schiff, PC = pulmonary cryptococcosis.

The characteristics of the patients with PC and without HIV are shown in Table 1. The number of patients with PC was 47 in total, including 45 definitive cases and 2 possible cases. Two of the 45 patients with confirmed PA gave body fluids from which *Cryptococcus gattii* was cultured (2, 4.4%). Of the patients without positive culture or histopathological evidence of *Cryptococcus* infection, 2 cases were diagnosed with probable cryptococcal infections and showed improvement with targeted antifungal treatment (Fig. 1).

**Table 1:** Characteristics of pulmonary cryptococcosis patients.

Variable	Patients with PC (%) (n = 47)
Male sex, no (%)	35 (88)
Age range (median)	20–71 (47)
Pulmonary symptoms	27 (57.4)
Sputum cultures	47
Positive	2 (4.4)
Negative	45 (95.6)
Disease history	18
Healthy	31 (65.9)
Organ transplantation	6 (12.8)
Diabetes	2 (4.3)
Nephrotic syndrome	2 (4.3)
Polymyositis	2 (4.3)
Solid tumor	2 (4.3)
Tuberculosis	1 (2.1)
Uremia	1 (2.1)
Use of glucocorticoids	6 (12.8)
Use of immunosuppressants	9 (19.1)

NO-PC = non pulmnoray cryptococcosis, PC = pulmnoray cryptococcosis.

In a total of 47 patients with PC, 27 patients (57.4%) had pulmonary symptoms, including cough, sputum, fever, chest pain, hemoptysis, and shortness of breath. Twenty-nine patients (61.7%) were previously healthy and 16 patients (34.0%) had a history of hypertension and severe diabetes mellitus with organ transplantation (6, 12.8%), diabetes (2, 4.3%), nephrotic syndrome (2, 4.3%), polymyositis (2, 4.3%), solid tumor (2, 4.3%), tuberculosis (1, 2.1%), uremia (1, 2.1%), glucocorticoid use (6, 12.8%), and immunosuppressant use (9, 19.1%).

### 3.2. CT findings

**Figure 2.**
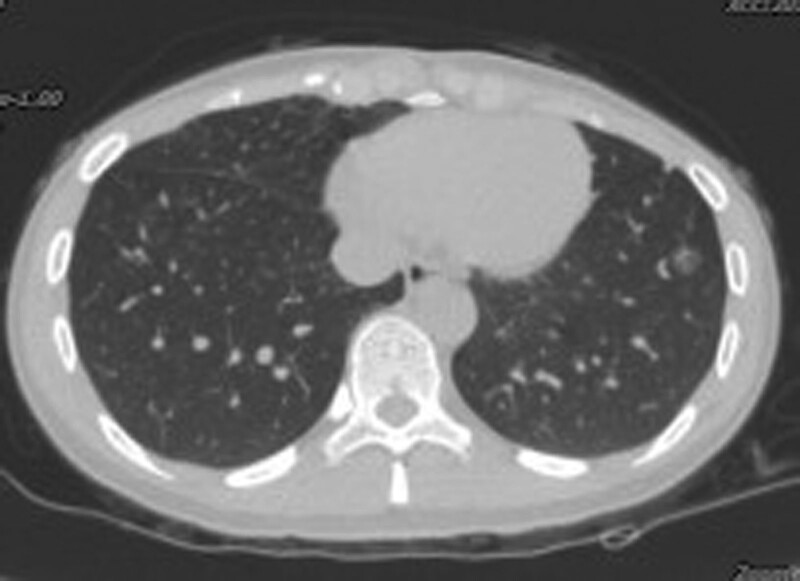
Chest CT scan of a novel false-negative PC patient. A 44-year-old nonsmoking female with a ground-glass opacity in the left lower lung showed false-negative CrAg LFA detection in the lung aspirate after CT-guided percutaneous lung biopsy. No suitable pathological tissue was obtained and the patient eventually underwent thoracoscopic surgery and was diagnosed with PC. CrAg = cryptococcal capsular polysaccharide antigen, CT = computed tomography, LFA = lateral flow immunoassay, PC = pulmonary cryptococcosis.

All 47 patients, except for 1 patient negative for CrAg in both serum and lung aspirate (Fig. 2), were categorized into the following 2 groups based upon the CrAg test results. Group I included 36 patients positive for CrAg in both the serum and lung aspirate and group II included 10 patients positive for CrAg in the lung aspirate, but not in the serum. The radiographic features of the patients in each group were assessed (Table 2).

**Table 2:** Correlation between the imaging and cryptococcal antigen testing results.

Variable	Group I (n = 36)	Group II (n = 10)	*P*
Distribution			
Single	7	6	.034
Unilateral multiple	11	2	.8
Bilateral multiple	18	2	.18
Morphological characteristics			
Nodule or mass	21	6	.79
Consolidation	4	3	.33
Mixed type	11	1	.37
Accompanying signs			
Vacuole or cavity sign	5	1	.84
Spiculation sign	4	2	.84
Pleura stretch sign	4	7	.00058
Halo sign	28	1	.00037
Air-bronchogram sign	12	1	.29
Bronchial truncation sign	9	0	.19

Group I, the patients who positive for CrAg in both the serum and lung aspirate; group II, the patients positive for CrAg in the lung aspirate but not in the serum.

The radiographic features of PC varied according to lesion shape. By analysis of the anatomical location of the lesions, peripheral or subpleural lesions were found in 44 cases (93.6%), intrapulmonary lesions in 1 case (2.1%), and diffuse lesions in 2 cases (4.3%) of all the 47 patients.

Of the 36 patients of group I, single (7, 19.4%), unilateral multiple nodules (11, 30.6%), and bilateral multiple nodules (18, 50.0%) were observed, while in the 10 patients of group II, single (6, 60.0%), unilateral multiple nodules (2, 20.0%), and bilateral multiple nodules (2, 20.0%) were observed. The results showed that the patients in group II had more single lesions than those in group I (*P* < .05).

There was no significant difference in the morphological characteristics, including nodule or mass, consolidation, or mixed type between the 2 groups (*P* > .05) (Table 2).

**Figure 3.**
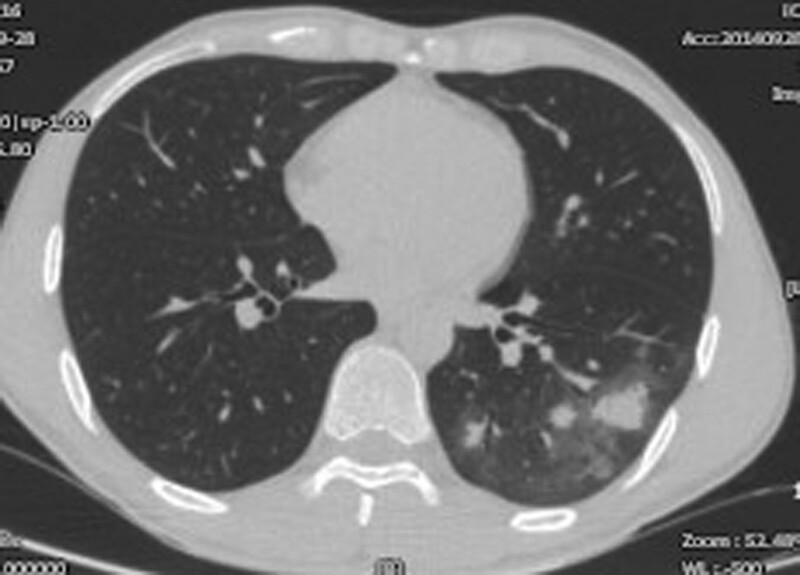
Representative case with pulmonary cryptococcosis showed some nodules accompanied by halo signs, positive for CrAg in both the serum and lung aspirate.

**Figure 4.**
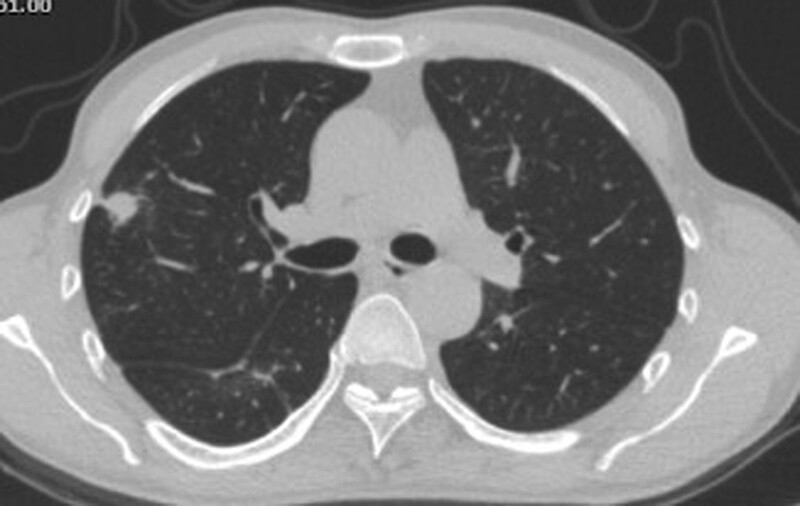
Representative case with pulmonary cryptococcosis showed a nodule accompanied by pleural stretch signs, positive for CrAg in the lung aspirate, but negative in the serum. The patient was confirmed to have PC by histopathological examination. CrAg = cryptococcal capsular polysaccharide antigen, PC = pulmonary cryptococcosis.

For concomitant signs, patients in group I showed more lesions accompanied by halo signs (*P* < .05) (Table 2 and Fig. 3), whereas those in group II showed more lesions accompanied by pleural stretch signs (*P* < .05) (Table 2 and Fig. 4).

### 3.3. Sensitivity and specificity of the CrAg test

The sensitivity and specificity of the CrAg test by LFA were analyzed in Table 3. The CrAg LFA test performed in the lung aspirate specimens of 46 of the total 97 patients were positive for *C. neoformans*, indicating 97.9% sensitivity, 100% specificity, 99% accuracy, positive predictive value of 100%, and negative predictive value of 98%. All 97 patients were also tested with the serum CrAg test. The results showed a substantially lower 76.6% sensitivity, 100% specificity, 88.7% accuracy, a positive predictive value of 100%, and a negative predictive value of 82%.

**Table 3:** Sensitivity and specificity of the cryptococcal antigen test by LFA.

Specimen	No. of patients positive for antigen/no. tested (%)							
	PC (n = 47)		NO-PC (n = 50)		Sensitivity	Specificity	PPV	NPV
	+	–	+	–				
serum	36	11	0	50	76.6	100	100	82
Lung aspirate	46	1	0	50	97.9	100	100	98

LFA = lateral flow immunoassay, NO-PC = non pulmnoray cryptococcosis, NPV = negative predictive value, PC = pulmnoray cryptococcosis, PPV = positive predictive value.

## 4. Discussion

PC is not rare in China; however, due to its nonspecific symptoms, the misdiagnosis rate is high.^[19]^ On account of the potential risk of central nervous system damage, high mortality, and long duration of antifungal therapy that are implicated with cryptococcosis,^[2]^ it is necessary to either establish or rule out PC in a timely fashion. However, only a few reports have focused on the performance of PC diagnostic procedures so far, especially in non-HIV patients.^[9]^

Serum CrAg tests, histopathology, and culture of lung tissue play different roles in diagnosis of PC. The specific histopathologic findings in lung tissue and positive cultures are the gold standards for the definitive diagnosis of PC, however, the limitations of these procedures are acknowledged.^[9]^ In this study, the lung lesions of 44 of the 47 PC patients were located in peripheral or subpleural areas, indicating that histology is the important for confirming PC. Only 2 patients had positive cultures, indicating the complementary role of culture. As each method has a distinct role, 1 often needs to combine multiple methods in order to confirm diagnoses or reach a high level of clinical suspicion.

The principle of CrAg test is to check for CrAg in serum or body fluids.^[20]^ Many studies have reported the accuracy of LFA at detecting CrAg, mostly with serum specimens.^[21–24]^ However, the sensitivity of serum CrAg test in HIV-negative patients with isolated PC is about only 82%.^[25]^ Due to the morbidity and mortality of cryptococcosis, the diagnostic value of disease-associated body fluid-specific CrAg LFA should be investigated. Here, we assessed the LFA method in PC confirmation with lung aspirate, aiming at the higher diagnostic ability.

Previous reports have indicated that the rate of direct detection of CrAg in BALF was higher than the rate in serum samples, especially in patients with pulmonary lesion diameters ≤25 mm.^[12]^ However, most pneumococcal lesions of PC patients are located in peripheral or subpleural areas, causing adequate sampling with bronchoscopy more difficult. CT-guided percutaneous transthoracic needle aspiration was shown to be a useful and safe diagnostic method with high yield in PC.^[25]^ CT-guided aspiration may provide a diagnostic yield greater than that of bronchoscopy. After CT-guided aspiration, effective pathological tissues in all but 1 patient were obtained. It was suggested that CT-guided aspiration is an effective and relatively less complicated procedure, as only 9.27% (9/97) cases showed mild complications, 4.12% (4/97) developed minimal pneumothorax, and 5.15% (5/97) suffered from mild self-limited hemoptysis. The results of CrAg LFA test in lung aspirate presented the comparable specificity (100% vs 100%) but superior sensitivity (97.9% vs 76.6%) lung aspirate CrAg LFA has when compared with the serum equivalent. In addition, the semiquantitative assay about the titer of CrAg in both serum and lung aspirate were performed and compared at the beginning, whereas we found there is not much valuable to diagnose this disease during this process. Therefore such results haven’t been stated here.

In patients who were positive for CrAg in both serum and lung aspirate, pulmonary lesions were more likely to be distributed singly and were more often than not accompanied by halo signs. However, in patients where lung aspirate but not serum CrAg was positive, pulmonary lesions were more likely to be accompanied by pleural traction signs. One of the 47 patients with PC had a false-negative CrAg LFA lung aspirate result, which may have been due to the inadequate volume of lung aspirate.

There were 2 possible cases in patients whose histopathology suggested inflammatory granuloma. These patients had negative plasma CrAg, but positive lung aspirate CrAg. After receiving antifungal therapy, the lesions disappeared. In addition, none of them was infected with Trichosporon, which cross-reacts with CrAg. These results support the accuracy of PC diagnoses when confirmed or rules out by CrAg lung aspirate tests.

The present results indicate that testing lung aspirate samples for the presence of CrAg with LFA may be diagnostically useful, especially for patients without HIV with single pulmonary lesions or lesions accompanied by pleural stretch, as is consistent with the previous results.^[13]^ Although this test has not yet been approved by the US Food and Drug Administration for the determination of CrAg in lung aspirates, we suggest that it be included as an adjunctive test for the evaluation of patients with PC. This study proves that the direct determination of CrAg in lung aspirates through CT-guided percutaneous needle lung aspiration is useful for PC diagnosis. However, this retrospective study had some limitations, including information bias. Hence, a prospective multicenter study is necessary to further evaluate the performance of LFA CrAg test in lung aspirate samples. In addition, cryptococcosis, as a neglected tropical disease, pose a serious threat to people’s life and health in developing countries or low-income regions.^[26]^ Recent studies have found that the mAb 18B7 based immunochromatoghaphic strip test was a novel lateral flow immunochromatoghaphic strip test, which was reliable, reproducible, and cost-effective as a point-of-care immunodiagnostic test for cryptococcosis.^[27]^ This point is particularly important for proper management of cryptococcosis.

## 5. Conclusion

In non-HIV patients, LFA CrAg detection in lung aspirate samples showed higher diagnostic value than that in serum samples, especially in patients with single pulmonary lesions or those accompanied by pleural stretch. The direct LFA measurement of CrAg in lung aspirate might be a rapid and useful test for the diagnosis of PC.

## Author contributions

Q.H., X.L., X.Z., C.Z., and Cai. Z. contributed to the study concept, design, data collection and analysis. Q.H. wrote the manuscript. Z.Z. reviewed and edited the manuscript. L.X. was in charge of revising the article and of final approval of the manuscript prior to submission.
